# Microsatellite Evidence for High Frequency of Multiple Paternity in the Marine Gastropod *Rapana venosa*

**DOI:** 10.1371/journal.pone.0086508

**Published:** 2014-01-23

**Authors:** Dongxiu Xue, Tao Zhang, Jin-Xian Liu

**Affiliations:** 1 Key Laboratory of Marine Ecology and Environmental Sciences, Institute of Oceanology, Chinese Academy of Sciences, Qingdao, China; 2 University of Chinese Academy of Sciences, Beijing, China; Australian Museum, Australia

## Abstract

**Background:**

Inferring of parentage in natural populations is important in understanding the mating systems of a species, which have great effects on its genetic structure and evolution. Muricidae, a large group (approximately 1,600 species) of marine gastropods, are poorly investigated in patterns of multiple paternity and sperm competition based on molecular techniques. The veined Rapa whelk, *Rapana venosa*, a commercially important muricid species with internal fertilization, is an ideal species to study the occurrence and frequency of multiple paternity and to facilitate understanding of their reproductive strategies.

**Methodology/Principal Findings:**

We developed five highly polymorphic microsatellites in *R. venosa* and applied them to identify multiple paternity in 19 broods (1381 embryos) collected from Dandong, China. Multiple paternity was detected in 17 (89.5%) of 19 broods. The number of sires per brood ranged from 1 to 7 (4.3 on average). Of the 17 multiply sired broods, 16 (94.1%) were significantly skewed from equal paternal contributions, and had a dominant sire which was also dominant in each assayed capsule.

**Conclusions:**

Our results indicate that a high level of multiple paternity occurs in the wild population of *R. venosa*. Similar patterns of multiple paternity in the 2–6 assayed capsules from each brood imply that fertilization events within the body of a female occur mostly (but not entirely) as random draws from a “well-but-not-perfectly blended sperm pool” of her several mates. Strongly skewed distributions of fertilization success among sires also suggest that sperm competition and/or cryptic female choice might be important for post-copulatory paternity biasing in this species.

## Introduction

Parentage studies and family reconstructions have become increasingly popular for investigating a range of evolutionary, ecological and behavioral process in natural populations [1]. Due to difficulties of observing mating in the wild, parentage in natural populations is usually difficult to determine by field observations. With the advent and increased use of molecular techniques, parentage in a wide range of animal groups has been determined [2]–[5].

Theoretically, one or a few matings are sufficient for females to fertilize all eggs and maximize their potential reproductive abilities for one reproductive period, and mating often carries relatively high cost and potential danger, so females are likely to be sexually monogamous [6]. Contrary to this prediction, female multiple mating (polyandry), where females mate with two or more males within a single reproductive season, is a taxonomically widespread phenomenon [5]. The evolution of female promiscuity and multiple paternity can be promoted by direct benefits (e.g., courtship feeding, nuptial gifts, fertilization assurance, paternal care and stimulation of reproduction) and indirect (genetic) benefits (e.g., acquisition of good genes, higher offspring diversity and genetic compatibility) [6]–[10]. In addition, mate encounter rate has also proved to be an important factor influencing levels of multiple paternity based on theoretical and empirical studies [11]–[13]. Sperm competition, which results from polyandry, refers to the competitive process between the sperm from two or more males for the fertilization of a given set of ova, and is a process of sexual selection and the final mode of males’ competition [14].

Parentage and sperm competition studies in marine gastropods and hermaphroditic land snails based on microsatellite evidence have shown that polyandry is an extremely common phenomenon in gastropods [15]–[21]. In these studied species (*Littorina saxatilis*, *Busycon carica*, *Solenosteira macrospira*, *Aplysia californica*, *Arianta arbustorum, etc.*), multiply-fertilized individuals accounted for about 76%–100% among all of the studied females, and the number of sires per brood ranged from 1–23. Patterns of sperm competition are variable in these species, some have no male sperm precedence [18], [22], [23] whilst others have last male sperm precedence [16], [19].

Muricidae is a large group (approximately 1,600 members) of marine gastropods [24]. Species of muricids are dioecious with internal fertilization, and fertilized eggs are encapsulated in capsules to be spawned. However, patterns of multiple paternity and sperm competition based on molecular techniques are poorly investigated in muricid species. Therefore, studies of mating systems of muricid species will facilitate understanding of their reproductive strategies and provide more insights into the mechanisms underlying female multiple mating and sperm competition.

The veined Rapa whelk *Rapana venosa* (Gastropoda: Muricidae), one of the most commercially important gastropod species in Asia, is abundant in silty sand of the intertidal and subtidal zones along the coasts of China, Korea and Japan [25]. It is also a notorious invasive species in Europe and North America [26]. Mating and spawning occurs from May to early August under seawater temperature of 24–27°C [25], [27]. In a hatchery environment, females mate and spawn many times in a single reproductive season [25], [27]. During spawning, groups of eggs, after being fertilized one after another in a chamber (receptaculum seminis) just posterior to the pallial oviduct, are extruded into the latter where they became encapsulated [28], [29]. The total number of egg capsules laid per female is 184–410 and the mean number of eggs in an egg capsule is 976 [25]. As divers have observed, the adults of *R. venosa* herd together in the wild during the reproductive season (personal communications), which should increase the rate of mate encounter and may affect the level of multiple paternity.

In this study, we developed five highly polymorphic microsatellites in *R. venosa* to determine the occurrence of multiple paternity in 19 broods obtained from one wild population. This study allows addressing two main questions: 1) What is the frequency and degree of multiple paternity in the veined Rapa whelk? 2) How much reproductive skew exists among the sires of a brood?

## Materials and Methods

### Ethics Statement

The collecting of egg-case strings of *R. venosa* from the Big Deer Island of Dandong was permitted by Zongyi Zhang, manager of the Dandong Daludao Haixing Group Co. Ltd. Ethical approval was not applicable for this study because no endangered animals were involved. However, all handling of the egg-case strings was conducted in strict accordance with Animal Care Quality Assurance in China.

### Sampling

Egg-case strings (n = 19), which were anchored on rocks at one end at depths of 4 - 10 meters, were collected by fishery divers in July 2012 along the Big Deer Island (Dandong, China). The egg-case strings were raised in the laboratory at 25°C, which is a suitable temperature for egg development of *R. venosa*. Before embryos were hatched from the egg-case capsule, two to six capsules per brood were randomly selected and preserved at −80°C.

### Microsatellite Development

Microsatellites were isolated from a single specimen of *R. venosa* following an enrichment protocol described by Glenn and Schable [30]. Primers flanking the microsatellite repeat regions were designed using Primer Premier version 5.00 (PREMIER Biosoft International). Primers were optimized and checked for polymorphisms using a sample of 30 adults. The number of alleles (*Na*), observed (*H_O_*) and expected (*H_E_*) heterozygosity were calculated by using the Excel Microsatellite Toolkit [31]. Hardy-Weinberg equilibrium (HWE) and genotypic linkage disequilibrium (LD) tests were performed with GENEPOP 4.0 [32]. Expected exclusion probabilities for each locus and across all loci were calculated with GERUD 2.0 [33]. The presence of null alleles for each locus was checked using Micro-Checker ver. 2.2.3 [34]. A total of five highly polymorphic loci were chosen for this study.

### DNA Extraction and Microsatellite Genotyping

Sixteen embryos were randomly selected from each capsule for DNA extraction which was performed according to the following protocol: each embryo was incubated for 3 h at 56°C with 20 µl of lysis buffer (10 mM Tris-HCl PH8.3, 50 mM KCl, 0.5% Tween-20, 500 µg/ml proteinase K), followed by 15 min at 95°C [35]. Samples were then centrifuged at 3000 rpm for 2 min to pellet cellular debris.

Genotyping of microsatellites was conducted using fluorescently-labeled primers and an automated ABI3730 XL DNA Sequencer. Five microsatellite loci (TB19, QR43, R12, R13, RV11), were amplified following the PCR protocol described in [36]. PCR products were electrophoresed on an ABI3730 XL DNA sequencer with the LIZ-500 size standard (Applied Biosystems). Data were analyzed using GeneMarker v2.2 (SoftGenetics, State College, PA, USA). Probably because of low DNA quality, two of the loci (R12 and RV11) were not genotyped successfully in some families (Locus R12 for Family 2, 6, 9 and 10, Locus Rv11 for Family 1, 3, 4 and 15). These loci were excluded for the subsequent analyses of parentage for these families.

### Analysis of Parentage

Analysis of paternity was conducted using the software COLONY 2.0 [37], [38], which uses a maximum likelihood method to assign parentage and sibship groups and to reconstruct the genotypes of the unsampled parents. COLONY evaluates genetic parentage by partitioning offspring into full-sib groups according to likelihood scores assuming Mendelian segregation and no maximum limit on the numbers of contributing parents. Offspring genotypes and known maternal sibships were entered into COLONY. The program was set to estimate and update allele frequencies during the analysis. Six replicate analyses were conducted for each brood by using three different random number seeds and two genotyping error rates (0.01 and 0.02). The number of sires contributing to each female’s brood and the reproductive skew among males were determined from the COLONY output. The degree of reproductive skew was measured by the binomial skew index B [36], [39]. A value of zero implies a random distribution of offspring among sires, positive values indicate skew, and significant negative values imply an overly equal distribution of offspring. Significant levels of *B* were estimated by simulation with 10,000 permutations. All of the skew analyses were conducted by SKEW CALCULATOR 2003 (http://www.eeb.ucla.edu/Faculty/Nonacs/PI.htm). A linear regression test for a correlation between the sample size and the number of sires was performed to assess whether larger samples were more likely to contain offspring from more of the males with whom a female had mated. To assess whether there were possible differences in a sire’s relative paternity contributions to the assayed capsules in each brood, Fisher’s exact tests were performed.

## Results

### Characterization of Microsatellites

All five microsatellite loci were highly variable, displaying from 11 to 32 alleles per locus (Table 1). Observed heterozygosities were high for all loci and ranged from 0.851 to 1.000 (Table 1). No evidence of genotypic disequilibrium between loci was detected, and all loci were in Hardy-Weinberg equilibrium. The exclusion probability (under the neither parent known model) was high for each of the five microsatellites analyzed and the combined exclusion probability for all five or four loci was larger than 99.65% (Table 1). Micro-Checker did not find any evidence for the presence of null alleles at any locus.

**Table 1:** Characteristics of five microsatellite loci in a sample of 30 adult individuals of *Rapana venosa*.

Locus	Repeat motif	Primer sequence(5′-3′)	Sizerange (bp)	*Ta* (°C)	*Na*	*H_E_*	*H_O_*	Exclusion probability
TB19 -FAM	(GA)31(CAGA)4(CACACAGA)3	F: CATCACCCTTAGGCCACAAT	134–224	56	32	0.973	0.844	0.843
		R: ACCTTTCCAAGTATCCACGA						
QR43 -FAM	(CA)27AA(CA)5GA(CA)4	F: CTTGTATCATTCATTCAACCTT	240–288	54	23	0.955	1.000	0.785
		R: GTTTCTTTTGGCTTCTTGTT						
R12 -HEX	(GT)37	F: CCTACCAAAAGTCAAGAGT	91–145	52	25	0.959	1.000	0.799
		R: CCCTGTGGGATAAGTATTG						
R13 -HEX	(ATC)10	F: TGCATAATGGTCCGTGAC	295–319	58	11	0.834	0.867	0.589
		R: ACATTTGGACTTGGTGGA						
RV11 -TAMRA	(TCTG)6(TC)7(AC)42	F: CTGTCCTCCATCTACCATA	154–234	52	22	0.955	0.851	0.770
		R: AATCTATTCCCTCCTTCAT						
Over all loci	N/A	N/A	N/A	N/A	N/A	0.934	0.912	0.999

### Genetic Paternity

**Figure 1:**
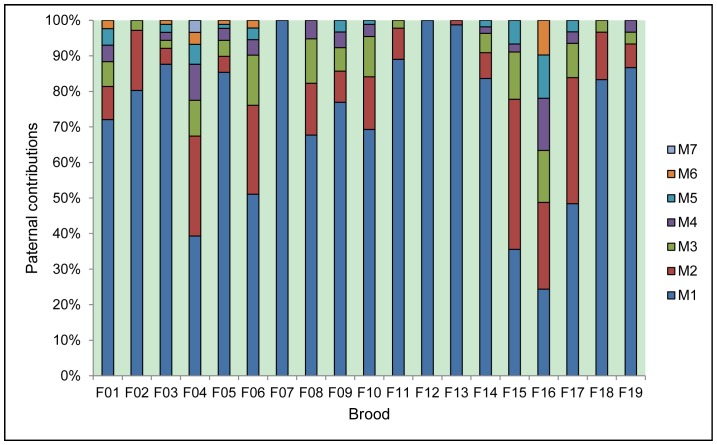
Relative contribution of sires within each brood of *Rapana venosa*.

Genotypes were obtained from 1381 of 1488 sampled embryos, representing progenies of 19 females. Assuming the same genotyping error rate (0.01 or 0.02), identical results were obtained for three replicate analyses with different random number seeds. Results of replicate analyses for two different genotyping error rates showed that high proportions of the offspring (94.80% - 98.91%) were assigned to identical full-sib families in all broods. Replicate analyses of 7 broods for the two different genotyping error rates gave identical results. However, replicate analyses of the other 12 broods showed a little variation: under the genotyping error rate of 0.01, one offspring in 7 broods and 2 to 4 offspring in 5 broods were assigned to 1 to 3 more full-sib families compared with results with the 0.02 error rate. The genotyping error rate was set to 0.02 in the downstream analyses as suggested by Wang [37]. For each brood, the best maximum likelihood inferred configuration consisting of full-sib families is shown in Table 1. Multiple paternity was detected in 17 (89.5%) of 19 broods. The number of sires per brood ranged from 1 to 7, with an average of 4.3 sires per brood (Table 2). Of the 17 multiply sired broods, 16 (94.1%) were significantly skewed from equal paternal contributions (Table 2, Figure 1), and had a dominant sire which was also dominant in each assayed capsule. Of 81 assayed capsules in the 17 multiply sired broods, 8 (9.9%) involved all the identified sires for the entire brood, and 9 (11.1%) only contained offspring from the dominant sire. The number of sires per brood was not significantly correlated with the sample size (*r* ^2^ = 0.01, df = 18, *P* = 0.80). For the 17 multiply sired broods, a total of 7802 Fisher’s exact tests was conducted for possible differences in a sire’s relative paternity contributions to the assayed capsules in each brood. Statistical significance (*P*<0.05) was reached in 7 (0.89%) of these comparisons. This number is much lower than that expected due solely to type I statistical error.

**Table 2:** Multiple mating for 19 broods of *Rapana venosa* from Dandong.

Family	Number ofanalyzed capsules	Number of genotyped offspring	Number of sires	M1	M2	M3	M4	M5	M6	M7	*B value*	*P*
F01	6	86	6	62	8	6	4	4	2		0.362	0.000
F02	6	71	3	57	12	2					0.331	0.000
F03	6	89	6	78	4	2	2	2	1		0.596	0.000
F04	6	89	7	35	25	9	9	5	3	3	0.107	0.000
F05	6	89	6	76	4	4	3	1	1		0.559	0.000
F06	6	92	6	47	23	13	4	3	2		0.171	0.000
F07	6	93	1	93							*NA*	*NA*
F08	6	96	4	65	14	12	5				0.240	0.000
F09	6	91	5	70	8	6	4	3			0.398	0.000
F10	6	88	5	61	13	10	3	1			0.307	0.000
F11	6	91	3	81	8	2					0.460	0.000
F12	6	94	1	94							*NA*	*NA*
F13	5	80	2	79	1						0.469	0.000
F14	4	55	5	46	4	3	1	1			0.494	0.000
F15	3	45	5	19	16	6	3	1			0.11	0.000
F16	3	41	6	10	10	6	6	5	4		0.000	**0.459**
F17	2	31	5	15	11	3	1	1			0.146	0.000
F18	2	30	3	25	4	1					0.358	0.000
F19	2	30	4	26	2	1	1				0.423	0.000

## Discussion

Parentage studies and family reconstructions in natural populations are a challenging endeavour, and obtaining accurate assignments is crucial to obtaining accurate representations of behavioral, ecological and evolutionary processes [1]. However, it is difficult to determine exactly how many microsatellite loci are necessary to achieve a certain level of power in sibship inference. For inferring parent numbers and individual parental contribution, high marker polymorphism appears to be much more important than solely increasing the number of makers with low genetic diversity [1], [40]. Although the accuracy of assignments increased with the number of loci [1], [40], Jones et al. [41] proposed to analyze no more than 3 or 4 loci in order to offset the costs associated with the genotyping of many broods. Based on analyses of simulated data, Sefc and Koblmüller [1], [40] suggested that COLONY performed well with 5 markers of *H*e = 0.84. In fact, 3 to 5 microsatellites have generally been used to reconstruct parentage for the majority of previous studies on gastropod species [15]–[21]. The average observed heterozygosity of the 5 microsatellite loci in the present study was 0.912, and the combined exclusion probability for all 5 or 4 loci was larger than 99.65%, which suggested that the microsatellite loci in our study could be sufficient to reconstruct parentage. Moreover, the presence of genotyping error had little impact on the accuracy of assignments [1], [37], and an analysis assuming an error rate of 0.01 gave essentially the same results as that assuming an error rate of 0.02 in our study.

Although there are over 1,600 living species in the family Muricidae [24], little is known about their genetic mating system in wild populations. The present study provides the first insights into the occurrence and frequency of multiple paternity in *R. venosa*, an commercially and ecologically important member in Muricidae.

Our findings, based on microsatellite assessments of 1381 embryos from 19 broods, indicate that a high level of multiple paternity (1 to 7, mean 4.3) occurs in the wild population of *R. venosa*. Since only a fraction of each brood was analyzed, the number of males contributing to each brood may be an underestimate of the true number of sires. The frequency of multiple paternity detected in *R. venosa* (89.5%) is low compared with some previously studied marine gastropods, such as *Littorina saxatilis* (100%) [42], *Solenosteira macrospira* (100%) [20], and *Busycon carica* (92%) [18], but higher than that of *Neptunea arthritica* (77%) and *Aplysia californica* (76%) [16]. Although there is no evidence that a *R. venosa* female does not obtain courtship feeding, nuptial gifts, or paternal care from multiple mating, we consider that the direct benefits obtained by a female from this behavior is more likely fertilization assurance. Females could also gain other fitness benefits such as the promotion of sperm competition, insurance against genetic abnormalities or inviable sperm from particular males and high genetic diversity among her progeny [18]. Furthermore, the high mate encounter rate of *R. venosa* during the reproductive season may be an important factor that leads to the high level of multiple paternity detected in *R. venosa.* Factors affecting male mate choice can also limit the level of female multiple mating. Males of some gastropods [19], [43], [44] have been reported to avoid the sperm competition risk by determining a female’s mating status using chemical cues or other cues.

In *R. venosa*, the male deposits sperm into the copulatory bursa to which the penis has access during mating. After copulation, sperm are transported from the bursa to the seminal receptacle where fertilization may occur. Our inability to reject the null hypothesis of relative paternity constancy in the assayed capsules from each brood suggests that sperm from multiple males should be mixed within the female’s reproductive tract. Thus, it is quite possible that fertilization events within the body of a female whelk occur mostly (but not entirely) as if by random draws from a “well-blended sperm pool” comprised of different ejaculate titers by her several mates [18]. Strongly skewed distributions of fertilization success among sires were detected in 94.1% of multiple mated females, suggesting that sperm competition and/or cryptic female choice might be important for post-copulatory paternity biasing in this species.

Different mechanisms of sperm competition exist in gastropod species. In the Neptune whelk, *Neptunea arthritiga*, males can achieve considerable reproductive success with a mated female through sperm removal, which implies last male sperm precedence [19]. However, in the garden snail, *Cornu aspersum* Müller, early sperm donors have been shown to sire more offspring by generating a resistance to incoming sperm cells through a unified beating of flagella of sperm residing in the female storage organ [45]. Results in the present study indicate that sperm competition may occur in *R. venosa*, however, it is impossible to elucidate the mechanisms of sperm competition of *R. venosa* due to the lack of the information on mating behaviors (e.g. mating order, copulation duration, intermating interval) and parental genetic information.

The post-copulatory female choice is another possible scenario that we can not exclude with the genetic data to explain the paternity biasing in *R. venosa*. It may be accomplished in a number of ways, such as sperm dumping, sperm digestion in female storage organs, or other means of differential sperm sorting and usage within the female reproductive tract [15], to determine the distribution of paternal contributions.

In the present study, only one wild population of *R. venosa* was analyzed. To fully understand the occurrence of sperm competition and cryptic female choice in this species, it would be very interesting to design experiments that track the sperm of individual male in the resulting offspring by observing their mating and spawning behaviors in laboratory and parentage analysis using genetic markers.

### Data Accessibility

A file containing the genotypes at 4 or 5 microsatellite loci of all offspring used in the Colony analyses is available from the Dryad Digital Repository: http://doi.org/10.5061/dryad.88741.
